# Validated determination of NRG1 Ig-like domain structure by mass spectrometry coupled with computational modeling

**DOI:** 10.1038/s42003-022-03411-y

**Published:** 2022-05-12

**Authors:** Niloofar Abolhasani Khaje, Alexander Eletsky, Sarah E. Biehn, Charles K. Mobley, Monique J. Rogals, Yoonkyoo Kim, Sushil K. Mishra, Robert J. Doerksen, Steffen Lindert, James H. Prestegard, Joshua S. Sharp

**Affiliations:** 1 Department of BioMolecular Sciences, University of Mississippi, University, MS USA; 2 Complex Carbohydrate Research Center, University of Georgia, Athens, GA USA; 3 Department of Chemistry and Biochemistry, Ohio State University, Columbus, OH USA; 4 Glycoscience Center of Research Excellence, University of Mississippi, University, MS USA; 5 Department of Chemistry and Biochemistry, University of Mississippi, University, MS USA; 6 Present Address: Analytical Operations Department, Gilead Sciences, Foster City, CA USA; 7 Present Address: Protein Discovery Department, Impossible Foods, Redwood City, CA USA

**Keywords:** Molecular modelling, Structural biology, Solution-state NMR, Proteomics

## Abstract

High resolution hydroxyl radical protein footprinting (HR-HRPF) is a mass spectrometry-based method that measures the solvent exposure of multiple amino acids in a single experiment, offering constraints for experimentally informed computational modeling. HR-HRPF-based modeling has previously been used to accurately model the structure of proteins of known structure, but the technique has never been used to determine the structure of a protein of unknown structure. Here, we present the use of HR-HRPF-based modeling to determine the structure of the Ig-like domain of NRG1, a protein with no close homolog of known structure. Independent determination of the protein structure by both HR-HRPF-based modeling and heteronuclear NMR was carried out, with results compared only after both processes were complete. The HR-HRPF-based model was highly similar to the lowest energy NMR model, with a backbone RMSD of 1.6 Å. To our knowledge, this is the first use of HR-HRPF-based modeling to determine a previously uncharacterized protein structure.

## Abstract (web summary)

A mass spectrometry-based method guides computational modeling for *de novo* protein structure prediction.

## Introduction

Mass spectrometry (MS) has rapidly gained in popularity not only in the identification and mass measurement of proteins, but in the characterization of protein higher order structure. Numerous MS-based technologies have been successfully used to characterize protein higher order structure, including hydrogen-deuterium exchange^1^, limited proteolysis^2^, chemical crosslinking^3^, and covalent labeling^4^. Covalent labeling includes a number of techniques, all of which involve reaction of some reagent with amino acid side chains usually available on the surface of the folded protein. A variety of covalent labeling reagents have been used, including acylation reagents^5^, diethylpyrocarbonate^6^, carbenes^7^, trifluoromethyl radicals^8,9^, and iodine radicals^10^. Here, we present an approach based on the use of hydroxyl radicals as a covalent labeling reagent. Hydroxyl radicals generate high-quality data for a variety of amino acids, providing a generalizable probe for protein topography^4,11–14^. We also demonstrate that this approach is capable of producing high-quality reliable protein structures that are validated in a blind test against a parallel determination by NMR methods.

The approach we use begins with data from a technique known as hydroxyl radical protein footprinting (HRPF)^15^. Hydroxyl radicals are useful and popular due to the wide variety of methods for in situ generation^16–23^, broad reactivity^13,14^, small size, hydrophilic nature, and well-characterized reaction pathways with various amino acids^24^. Work from Chance and co-workers found that apparent rates of reaction could be correlated with average solvent accessible surface area (<SASA>) once the inherent rate of reaction of the amino acid was corrected using the free amino acids as a surrogate^11,25^. Work from Sharp and co-workers confirmed these findings, further reporting that amino acids with lower inherent reactivity could display altered inherent reactivity based on sequence context^12,26^. Sharp and co-workers further used amino acid-resolution HRPF (known as HR-HRPF) coupled with computational modeling to demonstrate the ability to differentiate between accurate computational models and inaccurate computational models, opening possibilities for using HR-HRPF data to determine protein structure^12^.

HR-HRPF data are then used to facilitate computational predictions of structure. The Lindert group developed the first software to use covalent labeling data in automated Rosetta protein structure prediction^27,28^. Recently, Biehn and Lindert reported a more robust and less computationally expensive method for using HR-HRPF data to generate protein models using conical neighbor count instead of <SASA>, which successfully identified ab initio models of accurate atomic detail for three of the four benchmark proteins examined^29^. However, while these studies indicate the potential of HR-HRPF for the determination of protein structure, no protein of unknown structure has been determined using HR-HRPF data to inform computational modeling.

To accurately test the ability of HR-HRPF-based modeling to generate accurate novel protein structural models, we used the technology to determine the structure of the immunoglobulin-like domain (NRG1-Ig) of human neuregulin 1 (NRG1). NRG1 is a signaling glycoprotein that interacts with the ErbB/HER family of receptor tyrosine kinases via its EGF-like domain^30–32^. NRG1-mediated signaling plays an important role in neuronal and cardiac development, and regulation of synaptic plasticity^31–34^. Dysregulation of these signaling pathways is implicated in human disease, such as schizophrenia and certain forms of cancer^35,36^. Due to a combination of alternative splicing and proteolytic processing, NRG1 exhibits a high diversity of isoforms, both soluble and membrane-bound, and a number of these isoforms include the Ig-like domain^32,37^. In contrast to the EGF-like domain, the functional role of the 13.3 kDa NRG1-Ig domain is less well understood. It is believed to be involved in binding to heparan sulfate proteoglycans of the extracellular matrix^38,39^, and there are reports that it can affect ErbB receptor activation^40–42^.

In this manuscript, two teams worked independently to characterize the structure of NRG1-Ig. The first team used HR-HRPF to quantitatively measure topography of various amino acid side chains of the NRG1-Ig. Models of the protein were generated via Rosetta ab initio modeling, scored with the HRPF-guided Rosetta score term, then subjected to a Rosetta relaxation ensemble^29^ from which a top-scoring model was identified. Meanwhile, the second team determined the structure of NRG1 using standard heteronuclear solution NMR techniques. During structure determination, no data was shared between groups to prevent any bias. After both teams had generated their structural models, the HR-HRPF constrained structure was compared to the NMR structure, to assess the accuracy of the HR-HRPF method. The results of this study serve as a rigorous and unbiased test of the ability of HR-HRPF to facilitate a reliable determination of soluble protein structures.

## Results and discussion

### HR-HRPF of NRG1-Ig

NRG1-Ig was expressed in *E. coli* and purified as described in Supplementary Information and Fig. S1; structural homogeneity was verified by size exclusion chromatography and NMR. Proteolytic digestion of NRG1-Ig was optimized for maximum sequence coverage after complete digestion to maximize HR-HRPF data and reproducibility. GluC was found to generate considerably higher sequence coverage than trypsin (Fig. S2, Supplementary Information), with 98.3% of the NRG1-Ig sequence shown. GluC has also successfully been used in the past for HR-HRPF analysis, as the amino acids recognized by GluC are only minor oxidation targets^43^. Therefore, GluC was used for HR-HRPF analysis.

After purification and digestion optimization, multi-dose Fast Photochemical Oxidation of Proteins (FPOP)^12,22,44^ was performed on NRG1-Ig. For the purposes of this study, only FPOP data from native NRG1-Ig were used. A mixture of 10 µM NRG1-Ig, 17 mM glutamine, 1 mM adenine, 50 mM sodium phosphate, 2.2 mM Tris (pH 8.1), and hydrogen peroxide at 10 mM, 25 mM, 50 mM or 100 mM were used for FPOP labeling. Adenine dosimetry was measured for each experiment to determine delivered radical dose, in order to account for variability in radical generation or scavenging^45^. A control for each FPOP peroxide concentration was conducted under the same conditions without laser irradiation to measure and correct for background oxidation.

**Fig. 1:**
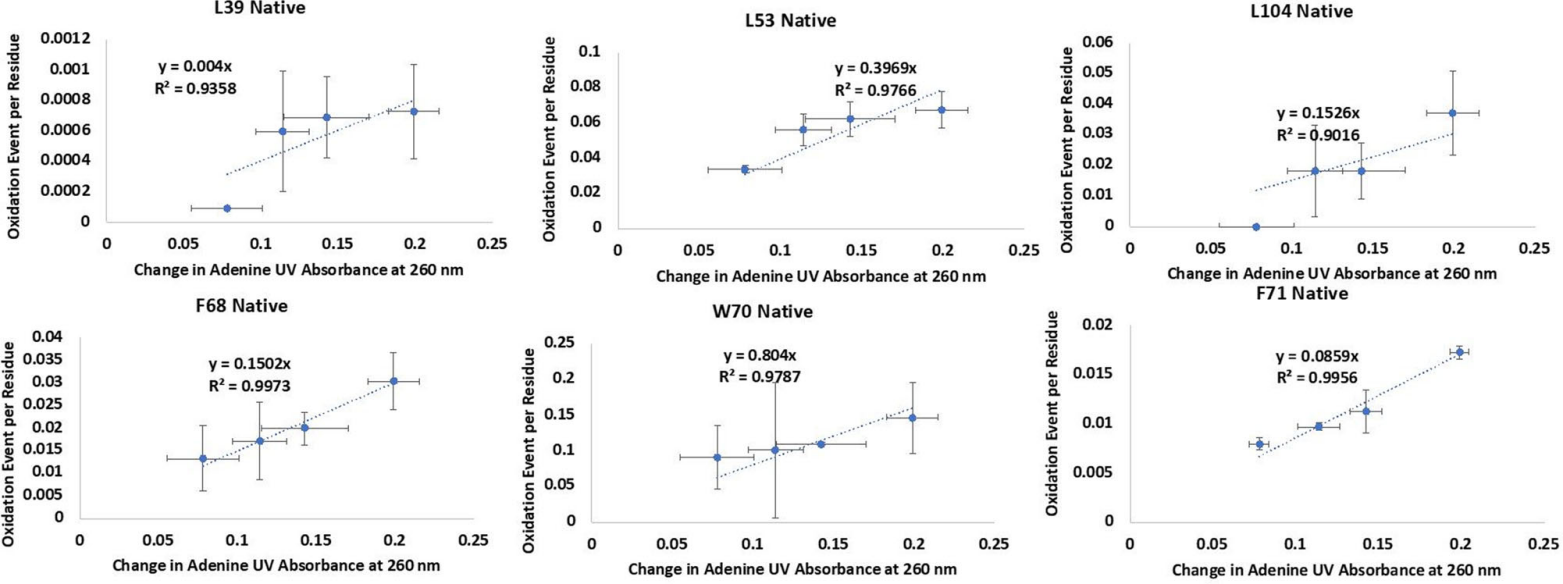
Measured radical dose response rate of six amino acids used for structural modeling in NRG1-Ig via HR-HRPF. Each figure shows the calculated oxidation of each residue at four different hydrogen peroxide concentrations plotted against the changes in adenine absorbance at 260 nm. The error bars represent one standard deviation from triplicate measurements for each data point. Each point represents the oxidation of one residue at a specific radical dose. The slopes of best-fit lines are radical dose responses.

Samples were then digested using our optimized GluC protocol. LC-MS/MS using electron transfer dissociation (ETD) was performed to measure the amount of oxidation at each amino acid for each oxidized peptide. Oxidation of twenty amino acids were measured (examples in Fig. 1, with full data in Fig. S3, Supplementary Information). Under FPOP conditions, the relationship between oxidation of the dosimeter and oxidation of a target residue will approach linearity, with the slope of the linear regression of the relationship being directly proportional to the reactivity of the oxidation target; a more complete explanation is given in Fig. S4, Supplementary Information. The slope of the regression was used to determine the protection factor (PF); 95% confidence intervals for slopes were used to represent uncertainty in PF measurement. PF was converted to the natural log of PF (lnPF), which was defined as the natural log of the normalized relative intrinsic reactivity value for a particular residue^14^ divided by the regression slope. Values measured for lnPF for all amino acids measured are given in Fig. S5 and Table S1, Supplementary Information.

### NMR analysis of NRG1-Ig

Using a suite of standard multidimensional experiments (Table S2, Supplementary Information), we obtained nearly complete resonance assigments of ^1^H, ^13^C, and ^15^N spins of the native polypeptide range (Table 1, Fig. S6, Supplementary Information). The only resonances we were unable to observe and assign were those of backbone ^1^H and ^15^N of Lys117. The ^13^C^α^ and ^13^C^β^ chemical shifts of the two cysteine residues were consistent with a disulfide bond formation^46^. Based on extensive chemical shift assignments and NOE data we obtained a well-defined solution NMR structure of NRG1-Ig (Fig. S7, Table 1, Supplementary Information). The fold of NRG1-Ig is typical of immunoglobulin-like domains, with a sandwich of two β-sheets stabilized by a disulfide bond. The smaller anti-parallel β-sheet consists of β-strands 41–58, 94–102, 86–91, while the second β-sheet consists of β-strands 77–72, 108–115, 120–130, 45–48 in a mixed topology with the last two stands running parallel. The only helical component is a single 3_10_ turn at 104–106.

**Table 1:** NRG1-Ig structure statistics.

Resonance assignment completeness^a^ [%]	
Backbone	99.6
Side-chain	100.0
Conformation-restricting distance constraints^b^	
Intra-residue [*i* = *j*]	367
Sequential [|*i* − *j*| = 1]	606
Medium-range [1 < |*i* – *j* < 5]	309
Long range [|*i* − *j*] ≥ 5	1150
Total	2432
Dihedral angle constraints (φ/ψ/χ_1_)	90/90/29
NOE constraints per restrained residue	24.0
Of those, long range	10.5
Average number of dihedral angle constraint violations per conformer > 10°	0
Average RMSD from mean coordinates^c^ [Å]	
Backbone atoms	0.5
Heavy atoms	0.9
Global quality scores^c^ (raw/*Z*-score)	
PROCHECK G-factor (phi-psi)	−0.59/−2.01
PROCHECK G-factor (all)	−0.37/−2.19
Molprobity clash score	5.64/0.56
ProsaII	0.25/−1.65
Verify3D	0.16/−4.82
Molprobity^3^ Ramachandran summary^c^ [%]	
Most favored regions	96.4
Additionally allowed regions	3.3
Disallowed regions	0.4
CYANA target function [Å^2^] (average over 20 conformers)	0.26
RPF analysis	
Recall/Precision/F-measure	0.959/0.918/0.938
DP-score	0.846

^a^Commonly observed protein NMR resonances. Excludes residues of the N-terminal purification tag, as well as side-chain amino groups of Lys, side-chain guanidinium groups of Arg, carboxyl groups of Asp and Glu, and hydroxyl ^1^H of Ser, Thr and Tyr, and non-protonated aromatic ^13^C.

^b^Calculated with PSVS v1.5.

^c^Ordered residue ranges: 34-61,66–132.

### Determining the best computational models of the Ig-like domain of NRG1

**Fig. 2:**
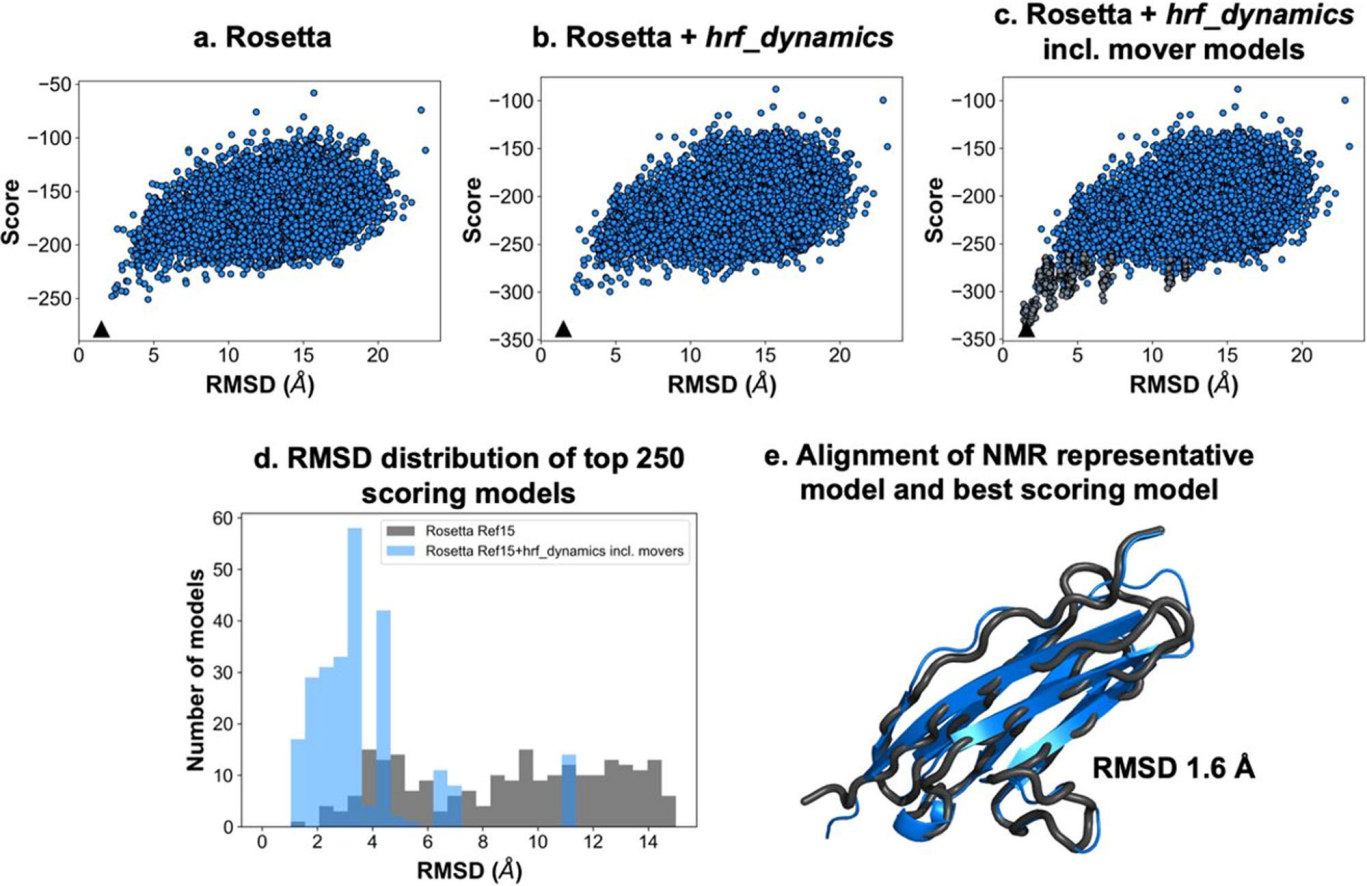
Ab initio modeling of NRG1-Ig with relaxation ensemble and FPOP-guided scoring significantly enriched high-quality models. Score versus RMSD to NMR model 1 when (**a**) scoring with Rosetta’s score function; (**b**) scoring with Rosetta and *hrf_dynamics*; and (**c**) scoring with Rosetta and *hrf_dynamics* including mover models (dark grey). Best scoring models are denoted by a black triangle. **d** RMSD histograms for top 250 scoring models when scoring with Rosetta (grey) versus Rosetta and *hrf_dynamics* including mover models (blue). Bin widths were maintained at 0.5 Å. **e** Alignment of NMR model 1 (black) with the top-scoring model identified from our HRPF-guided and mover model protocol (blue). The RMSD to the NMR model was determined to be 1.6 Å.

We employed our recent HRPF-guided Rosetta modeling protocol^29^ to predict the structure of NRG1-Ig. Sulfur-containing amino acids were excluded due to the prevelance of incompletely controlled secondary oxidation^12,47–49^. As per our published protocol, only lnPF values measured from Trp, Phe, Tyr, His, and Leu were used. Incorporation of other labeling targets increased the error observed between lnPF value and optimized conical neighbor count^29^. This is consistent with prior observation that the correlation between amino acid solvent accessible surface area and lnPF calculated using intrinsic reactivity values measured from free amino acids^14^ has much higher error as the intrinsic reactivity of the amino acid decreases, due to an increasing effect of the sequence context on the inherent reactivity of the amino acid^12^. Our protocol used an HRPF score term, *hrf_dynamics*, that rewarded models demonstrating agreement with the FPOP labeling data. The *hrf_dynamics* score term was previously developed based on the relationship between HRPF data and conical neighbor count, an exposure metric that is less computationally expensive to calculate than <SASA>. Based on its successful elucidiation of accurate models for three of four benchmark proteins, it was pursued for this work. Upon input of a user’s HRPF data as lnPF, the predicted neighbor count was calculated by substituting the lnPF value into the equation relating lnPF and conical neighbor count. Then, neighbor count was calculated for the input model to be scored, providing an observed neighbor count. The deviation between the observed and predicted neighbor count guides the scoring of the model, and models with predicted neighbor counts closer in value to observed neighbor counts were more rewarded. In this case, the rewarding process refers to receiving a more favorable, i.e., more negative, per-residue score. Upon scoring models with *hrf_dynamics*, we used Rosetta relaxation ensemble movers to sample protein flexibility. The output structures from the Rosetta mover protocol were referred to as mover models. Upon generation of 20,000 Rosetta ab initio models, we scored models with Rosetta’s score function (“Ref15”) (Fig. 2a) and *hrf_dynamics* to determine a total score (Fig. 2b). The 20 top-scoring models were then used as inputs for the relaxation ensemble that generated thirty mover models per top-scoring structure, leading to the addition of 600 models to be included in the model distribution (Fig. 2c).

**Fig. 3:**
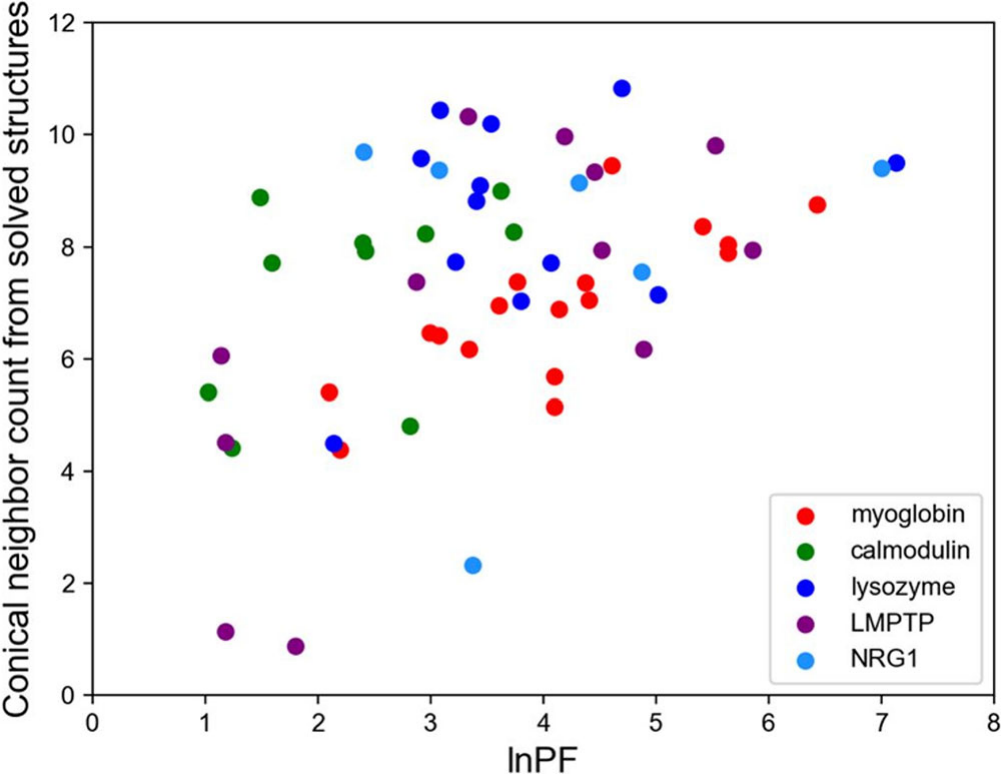
Correlation between HR-HRPF lnPF and conical neighbor count. The correlation measured for NRG1 performed blinded to the NMR structure (cyan) was consistent with those reported for proteins of known structure (red: myoglobin; green: calmodulin; blue: lysozyme; violet: LMPTP).

Upon examination of the 250 top-scoring models when scoring with Rosetta versus scoring with Rosetta and *hrf_dynamics* including mover models, we observed a decrease in the average RMSD and an increase in the percentage of models with RMSDs under 5 Å (Fig. 2d). The average RMSD of the top 250 models when scoring with Rosetta was 9.5 Å, which improved to 3.8 Å when scoring with FPOP data and including mover models. When scoring with Rosetta, 21% of the top 250 models had RMSDs below 5 Å. This improved with *hrf_dynamics* usage and mover model generation to 94% of models having RMSDs under 5 Å. When scoring both mover models and ab initio structures with our score term, we identified one of the generated mover models as the best scoring model. Our best scoring model exhibited an RMSD of 1.6 Å to the determined NMR structure of NRG1-Ig (Fig. 2e). The correlation between the HR-HRPF lnPF results for NRG1-Ig optimized conical neighbor count (the number of neighbors within the vicinity of a residue based on distance and angle contributions)^29^ from the lowest energy NMR structure was consistent with correlations previously reported for model protein structures^11,12,50^. The subset of amino acids considered here are robust regardless of the method of hydroxyl radical generation or amino acid-level quantitation, and no bias was introduced due to over-fitting to known structures (Fig. 3). Overall, employment of the relaxation ensemble to generate mover models resulted in a significant enrichment of accurate, high-quality, low-RMSD models in this blind prediction effort. We concluded that usage of our FPOP-guided and relaxation ensemble method increased confidence in model selection for other structure prediction efforts.

Subsequent to our completion of the NMR structure and HR-HRPF-assisted Rosetta model of NRG1-Ig, an AlphaFold model of NRG1 was released^51,52^ which made a high confidence prediction for the Ig-like domain (UniProt Q02297). This is indeed a high-quality structure with a 0.85 Å RMSD of α carbons for residues 29-116 of our NMR structure, discounting the less ordered N and C termini of our his-tagged 118 residue construct (Fig. S8, Supplementary Information). We have applied our conical neighbor count scoring function to the AlphaFold model and find that it scores similarly to our NMR and HR-HRPF-assisted Rosetta model (Table S3, Supplementary Information). This underscores the utility of HR-HRFP data and our scoring procedures regardless of the method of structure prediction. It is important to note that not all proteins or regions of proteins can be predicted with confidence at this point in time; for example, AlphaFold predicts less than 30% of the 640 residue NRG1 structure with confidence (pLDDT > 70). While some of these regions are probably intrinsically disordered in free solution under most conditions^53^, experimental structural biology techniques including HR-HRPF-based modeling will continue to play a vital role in determining structure in the absence of confident AlphaFold predictions, as well as in testing structural models where predictions are less confident.

## Conclusion

In this work, we tested the ability of HR-HRPF combined with conical neighbor count computational modeling to generate accurate, reliable structural models of a protein of truly unknown structure, NRG1-Ig. We were able to greatly increase the reliability of Rosetta modeling by application of HR-HRPF data, generating a final model with a backbone RMSD of <2 Å from the lowest energy NMR model, and with a large increase in model reliability. As the NRG1-Ig structure was unknown when HR-HRPF was performed and the NMR structure was determined independent of the HR-HRPF group, we have excluded any possibility of confirmation bias in experimental design. The consistency of our results with previous work published on proteins of known structure shown in Fig. 3 reveals a lack of confirmation bias in these previous results, and indicate no clear difference in accuracy based on the method of radical generation or amino acid-level oxidation quantification for the subset of amino acids used here (Trp, Phe, Tyr, His and Leu).

Our results as independently confirmed in a blind study by established NMR techniques demonstrate that HR-HRPF combined with conical neighbor count computational modeling is not just a tool for examining relative changes in protein topography, but is a structural biology tool that generates experimentally informed computational models of protein structure that are accurate and reliable. With the rise in computational tools for structural prediction including the recently released AlphaFold^51,54^, there is a need for flexible experimental methods to validate predicted structures. HR-HRPF has no theoretical limitations on the size or dynamics of measured protein structures, and can be carried out using microgram quantities of protein. Given the flexibility and low sample requirements of HR-HRPF compared with traditional high-resolution structural biology techniques, this methodology can play a significant role in the validation of computational structures, as well as the generation of accurate and reliable structural models when computational methods fail. Future work examining the ability of HR-HRPF combined with conical neighbor count to correctly identify domain-domain contacts and orientation are important for developing the application of HR-HRPF combined with conical neighbor count to address challenging problems in multi-domain protein structural biology.

## Methods

### Materials

Methionine amide was purchased from Bachem (Torrance, CA, USA). Adenine and L-glutamine were obtained from Acros Organics (Geel, Belgium). Catalase, trifluoroacetic acid (TFA), TCEP, and sodium phosphate were purchased from Sigma-Aldrich (St. Louis, MO). LC-MS grade formic acid, LC/MS-grade acetonitrile and water, sodium phosphate buffer, and hydrogen peroxide (30%) were obtained from Fisher Scientific (Fair Lawn, NJ, USA). Dithiothreitol (DTT) was purchased from Soltec Ventures (Beverly, MA). Sequencing-grade modified trypsin and GluC was purchased from Promega Corp (Madison, WI). Purified water (18 MΩ) was obtained from an in-house Milli- Q purification system (Millipore, Billerica, MA, USA).

### Expression and purification for NMR studies

A pET-21b(+) plasmid containing a TEV-cleavable N-terminal His-tag and a 100 residue fragment comprising the NRG1-Ig domain (residues 34-133 of the UniProt Q02297 sequence) was purchased from GenScript (US distribution, Piscataway, NJ). This plasmid was transformed into BL21(DE3) *E. coli* cells (New England Biolabs, Ipswich, MA) using standard protocols. Transformed cells were applied onto LB agar plate with ampicillin followed by overnight incubation at 37 °C. A single colony was used to inoculate a 10 mL LB media with carbenicillin and incubated overnight at 37 °C. Cells were pelleted at 2000x g and resuspended in 3 mL of M9 media. Resuspended cells (600 µl) were used to inoculate a 50 mL M9 culture and incubated at 37 C until OD600 = 0.8. Transformed cell stock was prepared by pelleting a 5 mL aliquot, followed by resuspending in 600 µl LB and 300 ul glycerol and flash freezing with liquid nitrogen.

Glycerol stock was used to inoculate 10 mL of LB starter culture, followed by overnight incubation at 35 °C. Cells were then pelleted and resuspended in 1 L of LB medium, and incubated again at 35 °C. To produce NRG1-Ig at natural isotopic abundance expression in the 1 L culture was induced with 1 mM IPTG after reaching OD_600_ of ~0.6, with cells harvested 3 h after induction and frozen. For stable isotope-labeled samples, the 1 L LB culture was instead pelleted upon reaching OD_600_ of ~0.8, and the cell pellet was resuspended in 0.5 L of M9 minimal media containing ^15^NH_3_Cl with either ^13^C-glucose or 5% ^13^C-glucose. Incubation continued for about 1 hr at 35 °C when expression was induced with 50 µM IPTG. Cells were harvested after ~3 h by centrifugation and frozen.

Thawed cells were resuspended in lysis buffer (20 mM Tris pH 8.1, 300 mM NaCl, and 1 mM TCEP with protease inhibitors) at 4 °C and lysed using a French-press. The resulting lysate was centrifuged, and the pellet fraction containing inclusion bodies was resuspended in denaturing buffer (6 M Urea, 300 mM NaCl, 1 mM TCEP, 6 mM imidazole and 20 mM Tris pH 8.1) at 4 °C using either handheld or electric tissue homogenizer. NRG1-Ig was purified under denaturing conditions via immobilized metal affinity chromatography (IMAC) using NGC system (Bio-Rad) equipped with a 10 mL Co-NTA column. Elution of NRG-Ig1 was accomplished with a linear gradient beginning with 3% Buffer A (6 M Urea, 20 mM Tris pH 8.1 at 4 °C, 300 mM NaCl, 1 mM TCEP) and ending with 100% Buffer B (6 M Urea, 20 mM Tris, pH 8.1 at 4 °C, 200 mM imidazole, and 300 mM NaCl). The recovered U-^15^N,^13^C and natural abundance NRG1-Ig fractions were sealed in membrane tubing (Spectrapor, 6–8 kDa) and refolded by dialysis at 4 °C in four steps against a refolding buffer (20 mM Tris pH 8.1 at 4 °C, 300 mM NaCl) The refolding buffer was supplemented with 0.1 mM DDT and 50 µM ethylenediaminetetraacetic acid (EDTA) for the first dialysis stem, and only 0.1 mM DTT for the second step. U-^15^N,5% ^13^C-labeled NRG-Ig was refolded in the same manner using 0.5 mL 3.5 kDa Slide-A-Lyser cassette (Pierce). U-^15^N,^13^C- and U-^15^N,5%-^13^C-labeled NRG1-Ig were subsequently exchanged into NMR buffer (20 mM sodium phosphate pH 6.5, 100 mM NaCl) using 0.5 mL Amicon micro concentrators. U-^15^N,^13^C NMR samples (~35 µL in 1.7 mm capillary NMR tube), NRG1-Ig NC(I), and NRG1-Ig NC(II), consisted of 0.45 mM and 2.0 mM NRG1-Ig, respectively, with 0.05% sodium aside, 4 µM sodium trimethylsilylpropanesulfonate (DSS) and 7% D_2_O. U-^15^N,5% ^13^C NMR sample, NRG1-Ig NC5 (~40 µL in 1.7 mm capillary NMR tube), was prepared in the original TRIS refolding buffer, with 0.05% sodium aside, 5 µM sodium trimethylsilylpropanesulfonate (DSS) and 7% D_2_O.

### NRG1-Ig purification by size exclusion chromatography for HRPF studies

A protein batch prepared by the NMR lab without isotope labels was run on Waters BEH SEC Column, 125 Å, 1.7 µm, 4.6 mm*300 mm using Thermo Fisher Dionex 3000 HPLC system. The running buffer was 20 mM Tris at pH 8.1 with 300 mM NaCl using an isocratic gradient.

### Multi-dose FPOP and NRG1-Ig digestion

FPOP was performed in triplicate for three aliquots of NRG1 using a 248 nm COMPex Pro 102 high pulse energy excimer KrF laser in the presence of various hydrogen peroxide concentration (10 mM, 25 mM, 50 mM, and 100 mM)^12^. The experiment was done in triplicate for each hydrogen peroxide concentration. For FPOP on native NRG1, samples were prepared by mixing NRG1 to the final concentration of 10 μM in 50 mM sodium phosphate, 17 mM glutamine, and 1 mM adenine as a radical dosimeter^45^. Freshly prepared hydrogen peroxide at four different concentrations (10 mM, 25 mM, 50 mM, and 100 mM) was added to each sample prior laser exposure. A total volume of 20 μl of sample flowed through the excitation capillary at 17.34 µl/min. The nominal laser fluence at the plane of the excitation capillary was at 9.82 mJ/mm^2^ with 15% exclusion volume. After the laser irradiation, the samples were quenched in 25 ul quenching buffer containing 50 nM catalase and 20 mM methionine amide. The control sample for each hydrogen peroxide concentration was done in triplicate with the laser turned off. After laser exposure, we measured the changes in adenine UV absorbance of each oxidized sample as compared to each control at 265 nm using a nanodrop spectrophotometer. This represents the effective radical dose delivered to the protein^12^.

After quenching, the oxidized and control samples were denatured and reduced at 95 °C for 30 min in the presence of 5.5 mM DTT. After denaturation, the samples we put on ice for 2 min. More sodium phosphate buffer at pH 6 was added to keep its concentration at 30 mM prior to GluC addition. GluC was added in 1:20 enzyme:protein mass ratio. The samples were digested overnight for 14 h.

### C18 RPLC-MS/MS C18

LC-MS/MS was done using an Acclaim PepMap 100 C18 nanocolumn (0.075 mm × 150 mm, 2 µm particle size, 100 Å pore size, Thermo Fisher Scientific) coupled to a 300 µm i.d. ×5 mm C18 PepMap 100 trap column with 5 µm particle size (Thermo Fisher Scientific) to desalt and concentrate the samples before loading onto the C18 nanocolumn for separation. The capillary pump was used to load the samples onto the C18 trap column using buffer A (water + 0.05% TFA) and buffer B (acetonitrile + 0.05% TFA). We used a nanopump for chromatographic separation using mobile phase C (water + 0.1% formic acid) and mobile phase D (acetonitrile + 0.1% formic acid). Initially, the samples were loaded onto the C18 trap column in 2% B at 5 µl/min for 6 min. The trap column was then switched inline with the nanocolumn and trapped peptides were back-eluted onto the nanocolumn using the nanopump. Elution started by increasing solvent D in a linear gradient from 2% to 40% over 22 min. The gradient then ramped up to 95% D over 5 min and held isocratic for 3 min to wash the column. Buffer D was then decreased to 2% over 1 minute and held isocratic for 6 min to re-equilibrate the column for the next run. The samples were eluted directly into a nanospray source of a Thermo Fusion Tribrid orbitrap, where the spray voltage was set at 2600 V and ion transfer tube temperature at 300 °C. A full MS scan was obtained from 150 to 2000 *m/z*. CID and ETD was performed every 2 s on precursor ions of +2 charge and greater for peptide identification and sequence coverage analysis. For ions with +2 charge state, ETD was performed with 20% EThcD SA collision energy to increase ETD fragmentation. The orbitrap resolution for both ETD and EThcD was 30,000 with AGC target at 5e4 and maximum injection time of 100 ms.

### Peptide and amino acid level oxidation analysis

Byonic version v2.10.5 (Protein Metrics) was used to identify NRG1 peptide sequences using the NRG1-Ig protein sequence described above. For all peptides detected, the major oxidation products detected were net additions of one or more oxygen atoms. In order to calculate average oxidation events per peptide, the area under the curve for peaks of unoxidized and oxidized peptides was used according to Eq. (1). Briefly, the oxidation events per peptide were calculated by summing the intensity (I) of each peptide oxidation product multiplied by the number of oxidation events on the peptide required to generate that product and divided by the sum of I for all oxidized and unoxidized versions of that peptide, as shown in Eq. (1) P represents the average oxidation events per peptide, and I is the area under the curve for peaks of oxidized and unoxidized peptides.

1 $$P=\left[\frac{{Isingly}\,{oxidi}{zed}* 1+I\,{doubly}\,{oxidized}* 2+I\,{triply}\,{oxidized}* 3+\ldots }{I\,{singly}\,{oxidized}+I\,{doubly}\,{oxidized}+I\,{triply}\,{oxidized}+\ldots }\right]$$

The amount of oxidation at residue level quantitation in a peptide was determined by the fragment ion (z or c ion) intensities of the peptide ETD fragmentation. The oxidation fraction of a given z or c ion was calculated by dividing the oxidized sequence ion intensity to the sum of the intensity of the corresponding oxidized and unoxidized sequence ion in a particular oxidized peptide. The relative oxidation fraction of each product ion f (*z*_*i*_) was calculated using **Eq. 3.2** where I(*z*_*i*_) is the intensity of the designated product ion, either summed across all spectra for RPLC, or taken at any individual point for ZIC-HILIC.

2 $$f\left({Zi}\right){relative}=\frac{I\left({Zi}\right){oxidized}}{\left(I\left({Zi}\right){oxidized}+I\left({Zi}\right){unoxidized}\right)}$$

The absolute amount of oxidation of a given amino acid was determined by multiplying the average oxidation event of peptide by the absolute fractional oxidation of the corresponding sequence ions. As shown in Eq. (3), P is the average oxidation event per peptide calculated from Eq. (1), and the term in brackets is the fractional difference of two adjacent sequence ions, f(*Z*_*i*_) and f(*Z*_*i*−1_). In cases where ETD fragmentation ions are not adjacent in sequence, fractional oxidation for multiple contiguous residues within the peptide can was calculated by using non-adjacent ETD fragments in Eq. (3).

3 $${Oxidation}\,{event}\,{per}\,{amino}\,{acid}=\left[f\left({Z}_{i}\right)-f\left({Z}_{i-1}\right)\right]* P$$

In order to take background oxidation into account, the oxidation event of each residue was calculated by subtracting the oxidation event of the same residue in control condition from its oxidation event in the oxidized sample.

Natural Protection Factor (ln(PF)) was calculated using Eq. (4) where ${R}_{i}$ represents the amino acid intrinsic reactivity for residue *i* while ${{Slope}}_{i}$ represents the experimentally determined radical dose response for residue *i*. Slopes with 95% confidence intervals were determined by linear regression analysis with the y-intercept constrained to zero.

4 $${ln}({PF}_{i})={Ln}\left(\frac{{R}_{i}}{{{Slope}}_{i}}\right)$$

### Structural modeling

Using Rosetta’s *AbInitioRelax* protocol, the NRG1 Ig-like domain FASTA sequence, and fragment libraries obtained from the Robetta server, 20,000 ab initio models of NRG1 were built^55–59^. No FPOP data were included during model generation. Models were scored with the Rosetta score function named “Ref15”. Per-residue FPOP data were converted into the natural log of the protection factor (lnPF), the natural log of the normalized intrinsic reactivity divided by the FPOP labeling rate constant^25,28,29^. The lnPF values were supplied to the *hrf_dynamics* term, and models were scored based on their agreement with the labeling data^29^. The summed per-residue *hrf_dynamics* score used a weight of 9.0, as described previously. The total score was determined by adding the Rosetta and *hrf_dynamics* scores. Models were ranked by total score. The twenty top-scoring models were then used as input for mover model generation with the Rosetta relaxation ensemble, as described previously^29^. For each of the top-scoring structures, thirty mover models were obtained. The six hundred mover models were scored with Rosetta and *hrf_dynamics* and then included in the ab initio model distribution. The best scoring model was identified as our blind prediction for the NRG1 Ig-like domain. Upon structure determination, Cα root mean squared deviation (RMSD) values with no outlier rejection were calculated with Rosetta.

### NRG1-Ig NMR

NMR data collection and processing, resonance assignment, and structure calculation followed the protocols of Northeast Structural Genomics Consortium (NESG Wiki, http://www.nmr2.buffalo.edu/nesg.wiki/Main_Page). NMR spectra (Table S2, Supplementary Information) for NRG1-Ig samples were acquired at 25 °C on AVANCE NEO 800 MHz spectrometer (Bruker BioSpin) equipped with a 1.7 mm TCI ^1^H(^13^C,^15^N) cryogenic probe. All spectra were Fourier-transformed using Topspin v4 (Bruker Biospin), except non-uniformly sampled 3D HBHA(CO)NH, which was reconstructed using Smile^60^ and Fourier-transformed with NMRPipe^61^. ^1^H chemical shifts were referenced relative to 4,4-dimethyl-4-silapentane-1-sulfonic acid (DSS), and ^13^C and ^15^N chemical shifts were referenced indirectly via gyromagnetic ratios. Visualization and analysis of NMR spectra, NOE peak picking, and integration were performed with the program CARA^62^. Automated assignment of backbone ^1^H, ^15^N, ^13^CO, ^13^C^α^, and ^13^C^β^ resonances was obtained with AutoAssign^63^ followed by interactive validation and completion. Side-chain resonances were assigned interactively using 3D (H)CCH and 3D ^13^C/^15^N-edited [^1^H,^1^H] NOESY spectra. Stereospecific assignments of Leu and Val isopropyl groups were obtained based on positive versus negative peak intensities in the 2D [^13^C,^1^H] constant-time HSQC (CT-HSQC) acquired for NRG1-Ig NC5, as described previously^64^. Stereospecific assignment of Asn and Gln CONH_2_ groups were determined from relative NOE peak intensities.

Structure calculation and automatic NOE peak assignment was performed iteratively using CYANA v 3.98.13^65,66^ and ASDP v1.0^67^. Constraints for backbone φ, ψ and side-chain χ_1_ dihedral angles were derived using TALOS-N^68^, and those that were consistent with the initial structural models were used in subsequent structure calculation steps. NOE peaks with matching unambiguous assignments from CYANA and ASDP were manually checked and refined for consistency with NOE spectra and distance constraint violations, and then used to optimize NOE distance calibration function. Assignments of these peaks were kept fixed during final structure calculation with CYANA. Stereospecific assignment of CH_2_ groups was performed iteratively using the GLOMSA module of CYANA. Of 100 calculated conformers, 20 conformers with the lowest target function values were further refined in explicit water bath using CNS^69^ as previously described^70^ with distance constraints relaxed by 5%. The quality of NRG1-Ig structure models was analyzed with PSVS^71^, and the resulting statistics are summarized in Table S3, Supplementary Information. Software used for NMR data analysis and structure calculation was accessed via NMRBox^72^. Atomic coordinates, structural restraints, assigned NMR chemical shifts, and NOE peaklists were deposited in the Protein Data Bank (PDB ID 7SJL) and BioMagResBank (accession code 30960).

### Reporting summary

Further information on research design is available in the Nature Research Reporting Summary linked to this article.

## Supplementary information

Supplementary Information

Reporting Summary

## Data Availability

Atomic coordinates, structural restraints, assigned NMR chemical shifts and NOE peaklists were deposited in the Protein Data Bank (PDB ID 7SJL) and BioMagResBank (accession code 30960). The datasets generated during and/or analyzed during the current study, including HR-HRPF LC-MS/MS data and the final HR-HRPF-based structural model presented, are available from the corresponding author on reasonable request.
