# Supplementary material for: Validated determination of NRG1 Ig-like domain structure by mass spectrometry coupled with computational modeling
Source: Commun Biol. 2022 May 12;5:452. doi: 10.1038/s42003-022-03411-y (PMC9098640; doi:10.1038/s42003-022-03411-y)
Supplement: Supplementary file 1 — Supplementary Information [file 42003_2022_3411_MOESM1_ESM.pdf]

Supplementary Information

Validated Determination of NRG1 Ig-like Domain Structure by Mass Spectrometry Coupled with Computational Modeling

Niloofar Abolhasani Khaje[1,a], Alexander Eletsky[2], Sarah E. Biehn[3], Charles K. Mobley[1,b], Monique J. Rogals[2], Yoonkyoo Kim[2], Sushil K. Mishra[1,4], Robert J. Doerksen[1,4] Steffen Lindert[3], James H. Prestegard[2], Joshua S. Sharp[1,4,5*]

1. Department of BioMolecular Sciences, University of Mississippi, University, MS 38677
2. Complex Carbohydrate Research Center, University of Georgia, Athens, GA 30602
3. Department of Chemistry and Biochemistry, Ohio State University, Columbus, OH 43210
4. Glycoscience Center of Research Excellence, University of Mississippi, University, MS 38677
5. Department of Chemistry and Biochemistry, University of Mississippi, University, MS 38677

a. Present address: Analytical Operations Department, Gilead Sciences, Foster City, CA 94404

b. Present address: Protein Discovery Department, Impossible Foods, Redwood City, CA 94063

* Corresponding author: jsharp@olemiss.edu

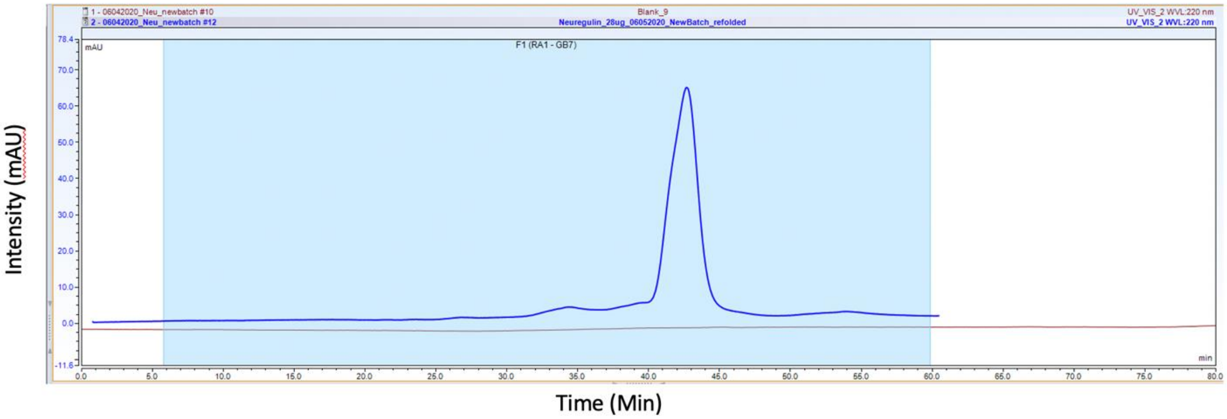

Figure S1. NRG1-Ig Analysis by SEC. Brown line represents the absorbance at 220 nm (A_{220}) of the blank run before running neuregulin. Blue line represents the A_{220} of NRG1-Ig, indicating the presence of one main conformation within the protein sample. Bottom-up MS/MS analysis of the major peak (retention time = 42-43 min) verified the identity as NRG1-Ig (data not shown).

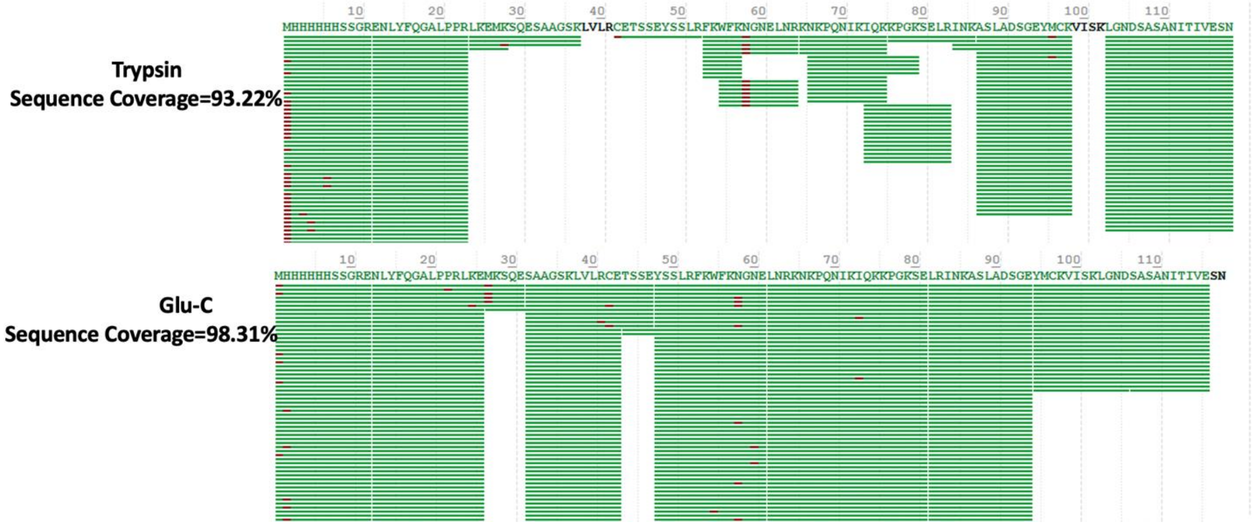

Figure S2. Sequence Coverage of Neuregulin using Trypsin and GluC enzymes. (A) Sequence coverage of neuregulin digested by trypsin. (B) Sequence coverage of neuregulin digested by GluC. Each horizontal bar represents an MS/MS spectrum assigned to the peptide covered by the width of the bar. Red spots represent chemical modifications to the peptide assigned by Byonic.

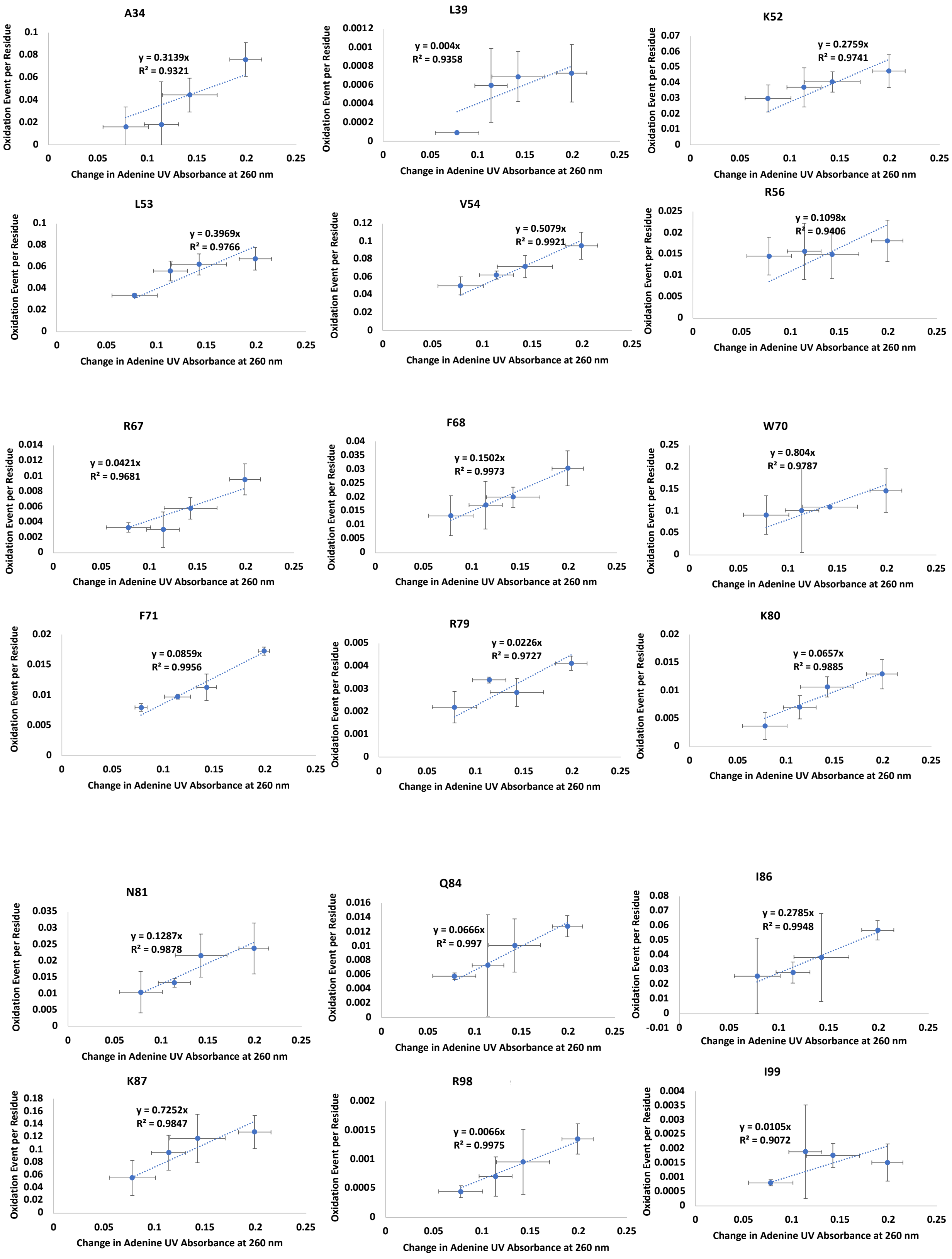
A34
y = 0.3139x
R² = 0.9321
L39
y = 0.004x
R² = 0.9358
K52
y = 0.2759x
R² = 0.9741
L53
y = 0.3969x
R² = 0.9766
V54
y = 0.5079x
R² = 0.9921
R56
y = 0.1098x
R² = 0.9406
R67
y = 0.0421x
R² = 0.9681
F68
y = 0.1502x
R² = 0.9973
W70
y = 0.804x
R² = 0.9787
F71
y = 0.0859x
R² = 0.9956
R79
y = 0.0226x
R² = 0.9727
K80
y = 0.0657x
R² = 0.9885
N81
y = 0.1287x
R² = 0.9878
Q84
y = 0.0666x
R² = 0.997
I86
y = 0.2785x
R² = 0.9948
K87
y = 0.7252x
R² = 0.9847
R98
y = 0.0066x
R² = 0.9975
I99
y = 0.0105x
R² = 0.9072
Oxidation Event per Residue
Change in Adenine UV Absorbance at 260 nm

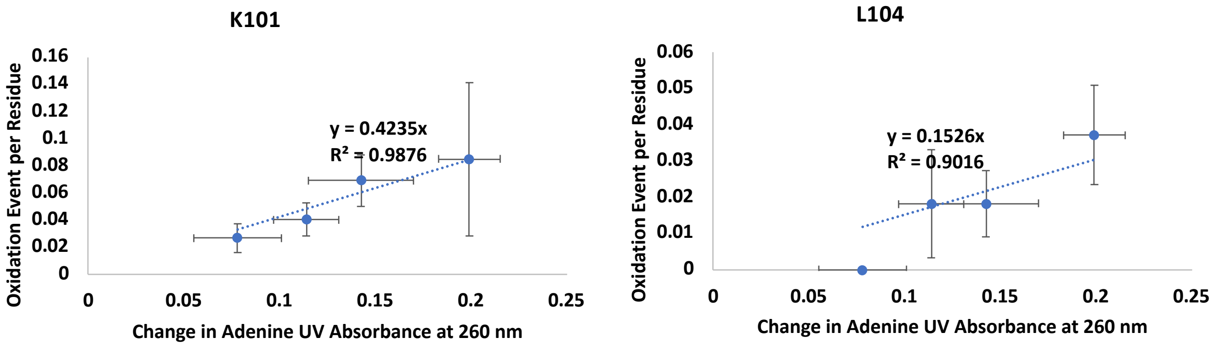

Figure S3. Measured radical dose response rate of all NRG1 amino acids in native form via HR-HRPF. Each figure is the calculated oxidation event of each residue at 4 different hydrogen peroxide concentrations plotted against the changes in adenine absorbance at 260 nm. The error bars represent one standard deviation from triplicate measurements for each data point. Each point represents the oxidation event of one each residue at a specific radical dose. The slope of this correlation is radical dose response.

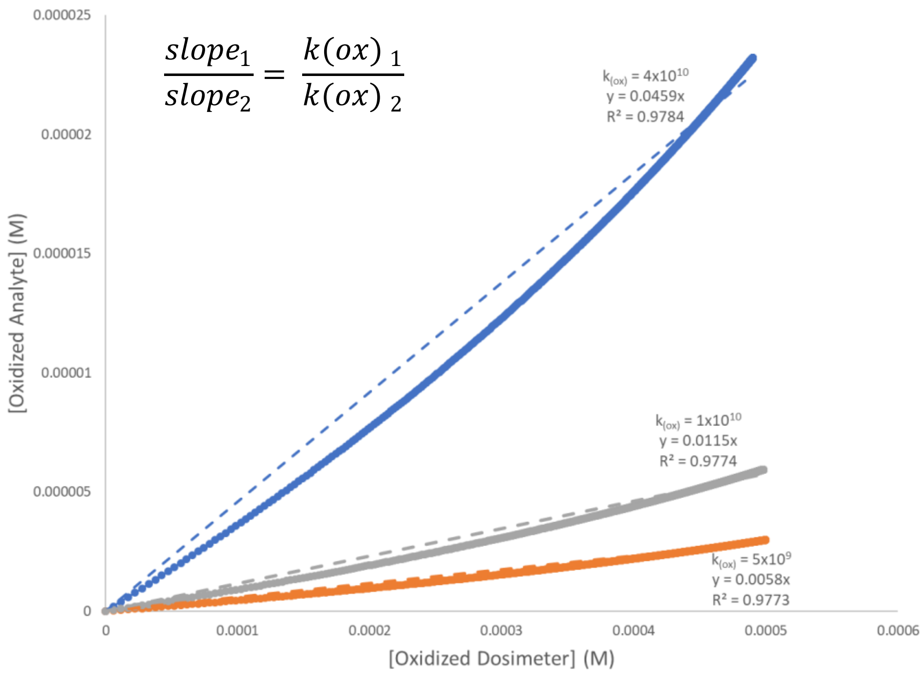

Figure S4. Theoretical relationship between dosimetry oxidation and analyte oxidation for analytes of different reactivities. Under FPOP conditions where dosimeter oxidation does not approach saturation, the theoretical relationship between dosimeter oxidation and amino acid analyte oxidation approaches linearity ($R^2 \approx 0.98$). Shown is the simulated relationship between dosimeter oxidation and amino acid analyte oxidation for three different categories of analyte: (blue) highly reactive analytes (pseudo-first order rate constant $k_{(ox)} = 4x10^{10}\ s^{-1}$), (grey) intermediate reactive analytes ($k_{(ox)} = 1x10^{10}\ s^{-1}$), and poorly reactive analytes ($k_{(ox)} = 5x10^{9}\ s^{-1}$). The slope of the linear regression is directly proportional to $k_{(ox)}$. Simulations were performed using Tenua. Competing reactions simulated were pseudo-first order oxidation of analyte with rate constant $k_{(ox)}$ (where oxidation of analyte does not consume analyte); second order oxidation of adenine dosimeter with rate constant $5.8x10^{9}\ M^{-1}\ s^{-1}$; and second order recombination of hydroxyl radical with rate constant $5.5x10^{9}\ M^{-1}\ s^{-1}$. Initial conditions were set to estimate those of FPOP: 1 mM ·OH, 5 µM analyte, and 1 mM adenine dosimeter.

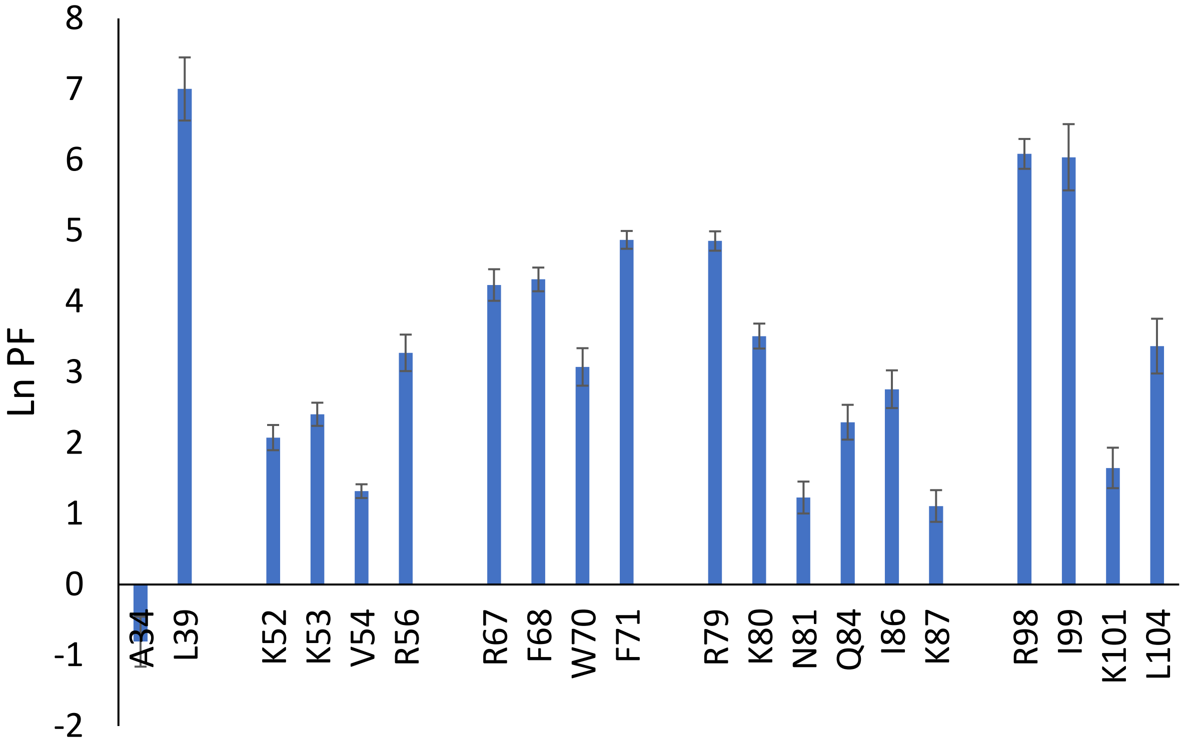

Figure S5. lnPF for each amino acid measured in NRG1. Error bars represent the 95% confidence interval based on linear regression analysis performed in **Figure 1** and **Figure S3**.

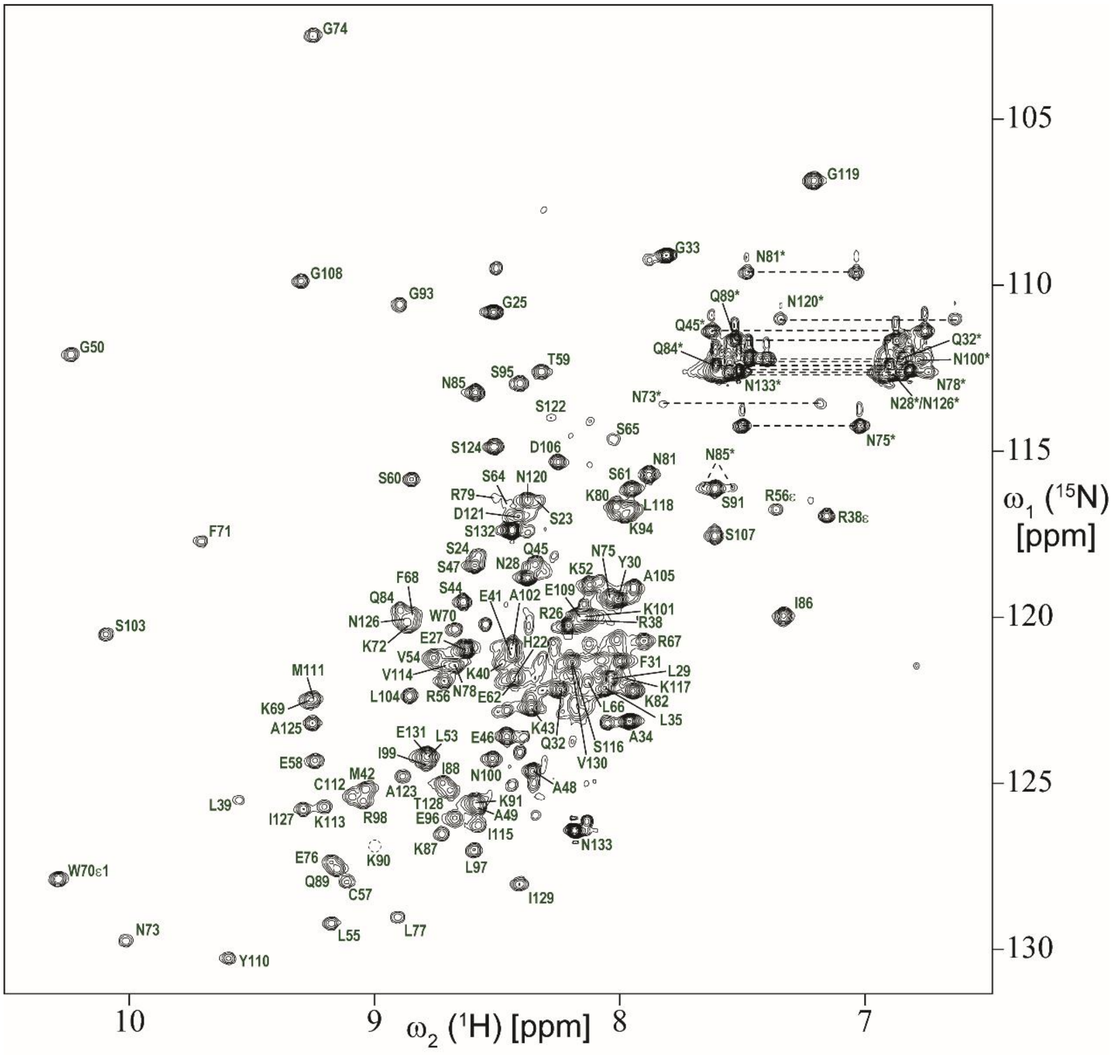

Figure S6. 2D [^{15}N, ^{1}H] HSQC spectrum of NRG1-Ig NC(II) sample acquired at 800 MHz magnetic field strength. Peak assignments are labeled in green with residue numbering corresponding to UniProt Q02297. Side chain resonances of Asn and Gln resonances are indicated with asterisks, also shown are side-chain resonances of Trp70 Hε1/Nε1, as well as Hε/Nε of Arg38 and Arg56. The latter two signals are 'aliased' into the observable region from their true ^{15}N chemical shift positions. Backbone amide signal of Lys90 is below the lowest displayed contour level, and its position is indicated by a dotted circle.

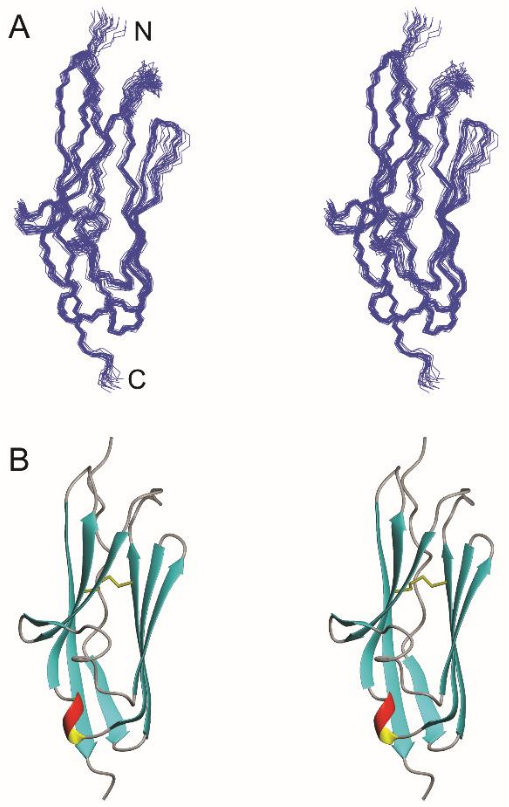

Figure S7. Stereo pair representation of solution NMR structure of NRG1-Ig. Only the native residues 34-133 are shown. **A.** Line plot of the 20 representative conformers superimposed for minimal RMSD of the C^{α} atoms of ordered residues. N- and C-termini are indicated. **B.** Ribbon diagram of the lowest-energy conformer showing the regular secondary structure elements. The disulfide bond linking the two β-sheets is shown as yellow sticks.

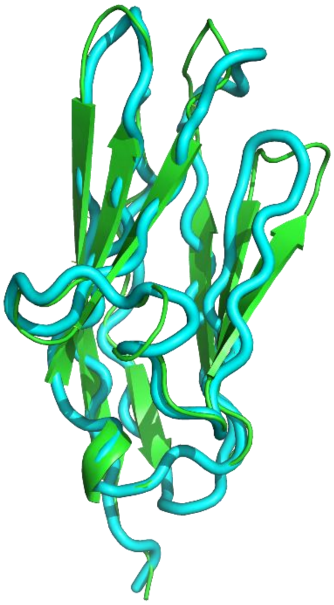

Figure S8. Alignment of (green) the AlphaFold 2 model of NRG1-Ig and (cyan) a representative NMR model of NRG1-Ig.

Table S1. lnPF for each amino acid measured in NRG1.

Amino Acid	Normalized Relative Intrinsic Reactivity[14]	lnPF ± 95% CI
A34	0.14	-0.81 ± 0.35
L39*	9.3	7.00 ± 0.45
K52	2.2	2.08 ± 0.18
L53*	9.3	2.41 ± 0.16
V54	1.9	1.32 ± 0.10
R56	2.9	3.27 ± 0.26
R67	2.9	4.23 ± 0.22
F68*	11.2	4.31 ± 0.17
W70*	17.4	3.07 ± 0.26
F71*	11.2	4.87 ± 0.13
R79	2.9	4.85 ± 0.14
K80	2.2	3.51 ± 0.18
N81	0.44	1.23 ± 0.23
Q84	0.66	2.29 ± 0.25
I86	4.4	2.76 ± 0.27
K87	2.2	1.11 ± 0.22
R98	2.9	6.09 ± 0.21
I99	4.4	6.04 ± 0.47
K101	2.2	1.65 ± 0.29
L104*	9.3	3.37 ± 0.39

***Amino acids used for *hrpf_dynamics* scoring**

Table S2. List of multidimensional NMR experiments used for resonance assignment and structure determination

Sample	Experiment
NRG1-Ig NC<br>2 mM	2D [^{15}N, ^{1}H] HSQC
	2D [^{13}C, ^{1}H] CT-HSQC (aliphatic)
	2D [^{13}C, ^{1}H] CT-HSQC (aromatic)
	3D (HACA)CONH
	3D HNCACB
	3D HBHA(CO)NH (NUS)
	3D (H)CCH-COSY aromatic
	3D (H)CCH-COSY aliphatic
	3D (H)CCH-TOCSY aliphatic,<br>16 ms mixing time
	3D ^{13}C/^{15}N-edited [^{1}H,^{1}H] NOESY, 80 ms mixing time
NRG1-Ig NC<br>450 µM	3D HNCO
	3D CBCA(CO)NH
NRG1-Ig NC5	2D [^{13}C, ^{1}H] CT-HSQC methyl, 28 ms CT delay

Table S3. Comparison of *hrf_dynamics* scores for NRG1-Ig models

**Model**	***hrf_dynamics* score**
AlphaFold 2 best ranked model	-56.812
HR-HRPF-assisted mover model	-59.932
NMR representative model	-57.557
