# Supplementary material for: Validated determination of NRG1 Ig-like domain structure by mass spectrometry coupled with computational modeling
Source: Commun Biol. 2022 May 12;5:452. doi: 10.1038/s42003-022-03411-y (PMC9098640; doi:10.1038/s42003-022-03411-y)
Supplement: Supplementary file 2 — Reporting Summary [file 42003_2022_3411_MOESM2_ESM.pdf]

nature portfolio

Corresponding author(s): Joshua S. Sharp

Last updated by author(s): 2022/03/29

Reporting Summary

Nature Portfolio wishes to improve the reproducibility of the work that we publish. This form provides structure for consistency and transparency in reporting. For further information on Nature Portfolio policies, see our Editorial Policies and the Editorial Policy Checklist.

Statistics

For all statistical analyses, confirm that the following items are present in the figure legend, table legend, main text, or Methods section.

n/a	Confirmed	
☐	☒	The exact sample size (*n*) for each experimental group/condition, given as a discrete number and unit of measurement
☐	☒	A statement on whether measurements were taken from distinct samples or whether the same sample was measured repeatedly
☐	☒	The statistical test(s) used AND whether they are one- or two-sided<br>*Only common tests should be described solely by name; describe more complex techniques in the Methods section.*
☒	☐	A description of all covariates tested
☒	☐	A description of any assumptions or corrections, such as tests of normality and adjustment for multiple comparisons
☐	☒	A full description of the statistical parameters including central tendency (e.g. means) or other basic estimates (e.g. regression coefficient) AND variation (e.g. standard deviation) or associated estimates of uncertainty (e.g. confidence intervals)
☒	☐	For null hypothesis testing, the test statistic (e.g. *F*, *t*, *r*) with confidence intervals, effect sizes, degrees of freedom and *P* value noted<br>*Give P values as exact values whenever suitable.*
☒	☐	For Bayesian analysis, information on the choice of priors and Markov chain Monte Carlo settings
☒	☐	For hierarchical and complex designs, identification of the appropriate level for tests and full reporting of outcomes
☒	☐	Estimates of effect sizes (e.g. Cohen's *d*, Pearson's *r*), indicating how they were calculated

Our web collection on statistics for biologists contains articles on many of the points above.

Software and code

Policy information about availability of computer code

Data collection	MS data were collected using Xcalibur. NMR data collection and processing, resonance assignment, and structure calculation followed the protocols of Northeast Structural Genomics Consortium (NESG Wiki, http://www.nmr2.buffalo.edu/nesg.wiki/Main_Page). NMR spectra (Table S2, Supplementary Information) for NRG1-Ig samples were acquired at 25 °C on AVANCE NEO 800 MHz spectrometer (Bruker BioSpin) equipped with a 1.7 mm TCI 1H(13C,15N) cryogenic probe. All spectra were Fourier-transformed using Topspin v4 (Bruker Biospin), except non-uniformly sampled 3D HBHA(CO)NH, which was reconstructed using Smile 59 and Fourier-transformed with NMRPipe.
Data analysis	Byonic version v2.10.5 (Protein Metrics) was used to identify NRG1 peptide sequences using the NRG1-Ig protein sequence. MS data were analyzed manually using QualBrowser. Using Rosetta's AbInitioRelax protocol, the neuregulin-1 Ig-like domain FASTA sequence, and fragment libraries obtained from the Robetta server, 20,000 ab initio models of neuregulin-1 were built. Models were scored with the Rosetta score function named "Ref15". Per-residue FPOP data were converted into the natural log of the protection factor (lnPF), the natural log of the normalized intrinsic reactivity divided by the FPOP labeling rate constant. The lnPF values were supplied to the hrf_dynamics term, and models were scored based on their agreement with the labeling data. The summed per-residue hrf_dynamics score used a weight of 9.0, as described previously. The total score was determined by adding the Rosetta and hrf_dynamics scores. Models were ranked by total score. The twenty top-scoring models were then used as input for mover model generation with the Rosetta relaxation ensemble, as described previously. For each of the top-scoring structures, thirty mover models were obtained. The six hundred mover models were scored with Rosetta and hrf_dynamics and then included in the ab initio model distribution. The best scoring model was identified as our blind prediction for the neuregulin-1 Ig-like domain. Upon structure determination, C? root mean squared deviation (RMSD) values with no outlier rejection were calculated with Rosetta. Visualization and analysis of NMR spectra, NOE peak picking and integration were performed with the program CARA . Automated assignment of backbone 1H, 15N, 13CO, 13C? and 13C? resonances was obtained with AutoAssign followed by interactive validation and completion. Side-chain resonances were assigned interactively using 3D (H)CCH and 3D 13C/15N-edited [1H,1H] NOESY spectra. Stereospecific assignments of Leu and Val isopropyl groups were obtained based on positive versus negative peak intensities in the 2D [13C,1H] constant-time HSQC (CT-HSQC) acquired for NRG1-Ig NC5, as described previously . Stereospecific assignment of Asn and Gln CONH2 groups were determined from relative NOE peak intensities.<br>Structure calculation and automatic NOE peak assignment was performed iteratively using CYANA v 3.98. and ASDP v1.0 66. Constraints for

backbone ?, ? and side-chain ?1 dihedral angles were derived using TALOS-N , and those that were consistent with the initial structural models were used in subsequent structure calculation steps. NOE peaks with matching unambiguous assignments from CYANA and ASDP were manually checked and refined for consistency with NOE spectra and distance constraint violations, and then used to optimize NOE distance calibration function. Assignments of these peaks were kept fixed during final structure calculation with CYANA. Stereospecific assignment of CH2 groups was performed iteratively using the GLOMSA module of CYANA. Of 100 calculated conformers, 20 conformers with the lowest target function values were further refined in explicit water bath using CNS as previously described with distance constraints relaxed by 5%. The quality of NRG1-Ig structure models was analyzed with PSVS , and the resulting statistics are summarized in Table S3, Supplementary Information. Software used for NMR data analysis and structure calculation was accessed via NMRBox . Atomic coordinates, structural restraints, assigned NMR chemical shifts and NOE peaklists were deposited in the Protein Data Bank (PDB ID 7SJL) and BioMagResBank (accession code 30960).

For manuscripts utilizing custom algorithms or software that are central to the research but not yet described in published literature, software must be made available to editors and reviewers. We strongly encourage code deposition in a community repository (e.g. GitHub). See the Nature Portfolio guidelines for submitting code & software for further information.

Data

Policy information about availability of data

All manuscripts must include a data availability statement. This statement should provide the following information, where applicable:

- Accession codes, unique identifiers, or web links for publicly available datasets
- A description of any restrictions on data availability
- For clinical datasets or third party data, please ensure that the statement adheres to our policy

The datasets generated during and/or analysed during the current study are available from the corresponding author on reasonable request.

Field-specific reporting

Please select the one below that is the best fit for your research. If you are not sure, read the appropriate sections before making your selection.

☒ Life sciences ☐ Behavioural & social sciences ☐ Ecological, evolutionary & environmental sciences

For a reference copy of the document with all sections, see nature.com/documents/nr-reporting-summary-flat.pdf

Life sciences study design

All studies must disclose on these points even when the disclosure is negative.

Sample size	Sample sizes wfor HR-HRPF were chosen based on sample availability and replicates typically used in the field.
Data exclusions	No data were excluded.
Replication	Replicates are described in the manuscript.
Randomization	Not applicable.
Blinding	The HR-HRPF modeling group and the NMR group were blinded regarding each other's structural model generated until both structural models were complete, as described in the manuscript.

Reporting for specific materials, systems and methods

We require information from authors about some types of materials, experimental systems and methods used in many studies. Here, indicate whether each material, system or method listed is relevant to your study. If you are not sure if a list item applies to your research, read the appropriate section before selecting a response.

Materials & experimental systems

n/a	Involved in the study	
☒	☐	Antibodies
☒	☐	Eukaryotic cell lines
☒	☐	Palaeontology and archaeology
☒	☐	Animals and other organisms
☒	☐	Human research participants
☒	☐	Clinical data
☒	☐	Dual use research of concern

Methods

n/a	Involved in the study	
☒	☐	ChIP-seq
☒	☐	Flow cytometry
☒	☐	MRI-based neuroimaging
